# Monitoring the Intracellular Tacrolimus Concentration in Kidney Transplant Recipients with Stable Graft Function

**DOI:** 10.1371/journal.pone.0153491

**Published:** 2016-04-15

**Authors:** Seung Seok Han, Seung Hee Yang, Min Chang Kim, Joo-Youn Cho, Sang-Il Min, Jung Pyo Lee, Dong Ki Kim, Jongwon Ha, Yon Su Kim

**Affiliations:** 1 Department of Biomedical Sciences, Seoul National University College of Medicine, Seoul, Korea; 2 Kidney Research Institute, Seoul National University, Seoul, Korea; 3 Department of Clinical Pharmacology and Therapeutics, Seoul National University College of Medicine, Seoul, Korea; 4 Department of Surgery, Seoul National University College of Medicine, Seoul, Korea; 5 Department of Internal Medicine, Seoul National University College of Medicine, Seoul, Korea; UNIFESP Federal University of São Paulo, BRAZIL

## Abstract

Although monitoring the intracellular concentration of immunosuppressive agents may be a promising approach to individualizing the therapy after organ transplantation, additional studies on this issue are needed prior to its clinical approval. We investigated the relationship between intracellular and whole blood concentrations of tacrolimus (IC-TAC and WB-TAC, respectively), the factors affecting this relationship, and the risk of rejection based upon IC-TAC in stable kidney recipients. Both IC-TAC and WB-TAC were measured simultaneously in 213 kidney recipients with stable graft function using LC-MS/MS. The tacrolimus ratio was defined as IC-TAC per WB-TAC. The genetic polymorphism of *ABCB1* gene and flow cytometric analyses were conducted to probe the correlation between tacrolimus concentrations and the immunoreactivity status as a potential risk of rejection, respectively. The correlation between IC-TAC and WB-TAC was relatively linear (*r* = 0.67; *P*<0.001). The factors affecting the tacrolimus ratio were sex, hematocrit, and the transplant duration, as follows: a high tacrolimus ratio was noted in female patients, patients with a low hematocrit, and patients with a short transplant period. However, the tacrolimus ratio did not reflect the prior clinical outcomes (e.g., rejection) or the genetic polymorphism of *ABCB1*. After stimulation with phorbol-12-myristate 13-acetate and ionomycin, the proportion of T cells producing interferon-gamma or interleukin-2 was higher in the low-IC-TAC group than in the high-IC-TAC group. Further studies are required to evaluate the value of the intracellular tacrolimus concentrations in several clinical settings, such as rejection, infection, and drug toxicity.

## Introduction

The annual rate of kidney transplantation has steadily increased owing to its proven survival benefit over other treatment options [1]. Although graft rejection is a major complication to recipients, the introduction of new immunosuppressive agents, such as calcineurin inhibitors, has led to a large reduction in the rejection rate and has increased the long-term utility of grafts [2]. The overall rate of acute rejection is approximately 10% within 1 year under the current immunosuppressive regimen [3]. However, this rate is suboptimal and should be further reduced because acute rejection *per se* is directly related to the economic burden of kidney transplantation as well as the overall graft outcome [4,5].

Previous evidence has confirmed that tacrolimus is the preferred choice of treatment for prolonging graft survival compared with other calcineurin inhibitors [6]. Furthermore, it is recommended that exposure to tacrolimus be reduced to a certain degree for the prevention of calcineurin inhibitor-induced nephrotoxicity [7]. Although there are global guidelines for the use of tacrolimus based on the immunological risk [8], detailed information about how to administer this drug to individual recipients under various circumstances has not been published. To reduce both the rejection and adverse events triggered by tacrolimus, the establishment of personalized guidelines for the use of tacrolimus is an essential step.

To accomplish this objective, several pharmacokinetic and pharmacodynamic methods have been suggested. In current pharmacokinetic monitoring protocols, adjustment of the dose of tacrolimus according to the whole blood level of tacrolimus is relatively simple and useful. However, this method alone is not enough to improve the patient’s outcome due to inter- and intra-individual differences in physiologic responses. In particular, it cannot be assumed that the tacrolimus concentration in whole blood is always linked to the tacrolimus concentration in lymphocytes, which are the targets of tacrolimus. Importantly, compared with the blood tacrolimus concentration, the intracellular tacrolimus concentration may be more closely related to the T cell responses against grafts [9]. However, data on the intracellular tacrolimus concentrations in kidney recipients are scarce. Moreover, previous studies in this area have focused only on the role of genetic polymorphisms with no regard for other factors [10]. To determine whether the measurements of intracellular tacrolimus concentration may be applicable to actual clinical practice, further studies on this issue are needed. Herein, we aimed to evaluate the relationship between whole blood and intracellular tacrolimus levels, the factors affecting this relationship, and the potential risk of rejection according to the intracellular level in kidney transplant recipients.

## Methods

### Ethics Statement

This investigation was conducted according to the principles expressed in the Declaration of Helsinki. All participants engaged voluntarily and signed informed consent forms. The institutional review board at the Seoul National University Hospital approved the study (H-1307-141-507).

### Participants

To the best of our knowledge, no studies have attempted to identify the factors affecting the balance between whole blood and intracellular tacrolimus levels in stable kidney recipients. One study revealed that genetic polymorphisms in the ATP-binding cassette subfamily B member 1 (*ABCB1*) were related to tacrolimus concentrations although no information was provided regarding the patients’ graft status or multivariate-adjusted results [10]. Based on this study result, we attempted to calculate the minimal sample size of the cohort. Using a two-sided 5% significance level, a power of 80%, an anticipated drop-out rate of 10%, and the minor allele frequency of rs1045642 (0.459), it was determined that a total of 210 patients was required. We screened patients prior to 4 weeks of tacrolimus measurement. The inclusion criteria were as follows: age ≥18 years, use of tacrolimus, stable graft function (change in serum creatinine of less than 0.3 mg/dL within 3 months), stable concentration of blood tacrolimus compared to the last concentration (i.e., there was no reason to alter the tacrolimus dose), and the ability and willingness to provide written informed consent. The exclusion criteria were as follows: multiple organ transplantation, change in the tacrolimus dose within 1 month of the inclusion assessment for any reason (e.g., rejection, infection, or decreased graft function), and the use of medications known to interact with tacrolimus and change the tacrolimus level (e.g., anti-fungal and anti-viral agents) [11]. Overall, 220 patients provided written informed consent. However, before or at the time of tacrolimus measurement, 6 patients were dropped out (withdrawal of consent (n = 4); change in graft function (n = 1); and no follow-up (n = 1)). Accordingly, 214 patients were enrolled between January 2014 and February 2015.

### Data collection

The clinical parameters recorded included the following: age; sex; donor source (living relative, living non-relative, or deceased); previous history of transplantation; diabetes mellitus; histories of delayed graft function, acute rejection, recurrent original disease, and calcineurin inhibitor-induced nephrotoxicity; and the duration of transplantation. Delayed graft function was defined as the need for dialysis within 1 week of transplantation. Other episodes (i.e., acute rejection, recurrence, and tacrolimus-induced nephrotoxicity) were defined as the occurrence of events within 6 months of transplantation. The patients used triple immunosuppressive agents, such as prednisolone, mycophenolate mofetil, and tacrolimus. The target range of WB-TAC in stable kidney recipients at our institute was between 2 and 8 ng/mL, although WB-TACs of seven patients were higher than 8 ng/mL. However, their doses of tacrolimus were not changed before and after the time of the study enrollment because of confirmed stable graft functions. Serum hematocrit, lymphocyte proportion, albumin, and creatinine were measured, and proteinuria was semi-quantitatively scored from negative to +4 with a dipstick test and defined as a score ≥+1. All of these baseline parameters were assessed when the tacrolimus level was examined.

The trough level of blood tacrolimus was measured by liquid chromatography/tandem mass spectrometry (LC-MS/MS) [Agilent 1260 Infinity Binary LC (Agilent Technologies, Santa Clara, CA, USA) and API 4000 QTRAP system (AB Sciex, Framingham, MA, USA)]. In parallel, the intracellular tacrolimus level was measured as follows. When the patients arrived at the center, peripheral blood mononuclear cells (PBMCs) were readily isolated from 8 mL of whole blood (collected in a heparinized tube) using a Ficoll gradient. The blood samples were centrifuged at 400×g for 40 minutes at 4°C. After collecting the PBMC layer, the cells were washed twice. Next, the viable cells were counted after excluding dead cells with trypan blue, using an automated cell counter (Invitrogen, Carlsbad, CA, USA). Cells were suspended in 1 mL of phosphate-buffered saline (PBS) and stored at -80°C until sample preparation for LC-MS/MS. All steps were conducted at 4°C to prevent the efflux of tacrolimus from the cells.

For LC-MS/MS sample preparation, 200 μL of suspended cells were mixed with 50 μL of internal standard (50 ng/mL ascomycin in 50% methanol) and 1 mL of methyl tertiary butyl ether. After vortexing and centrifugation (14,000 rpm, 5 minutes, 4°C), the organic solvent layer was dried using a nitrogen concentrator. The extract was reconstituted in 75 μL of 50% methanol and 0.1% formic acid. After vortexing and centrifugation under the same conditions, 5 μL of the reconstituted sample was injected into the LC-MS/MS. The LC-MS/MS instrumentation is described in S1 Instrumentation for LC–MS/MS. In addition to the whole blood and intracellular concentrations of tacrolimus (WB-TAC and IC-TAC, respectively), we defined the tacrolimus ratio as IC-TAC/WB-TAC (unit: pg.10^-6^ cells/ng.mL^-1^) to identify the cases with an unbalanced distribution of tacrolimus between whole blood and intracellular compartments. The assay development and validation processes were conducted based on the previous study results [10,12].

### Genotyping analysis

Genomic DNA was extracted from whole blood samples using the QIAamp DNA blood kit (Qiagen, Valencia, CA, USA) according to the manufacturer’s protocol. The determination of single nucleotide polymorphisms (SNPs) in *ABCB1* was performed using the SNaPshot Multiplex kit (Applied Biosystems, Foster City, CA, USA) according to the manufacturer’s instruction. The analysis was carried out using GeneMapper software (version 4.0; Applied Biosystems, Foster City, CA, USA). S1 Table lists the primer sets and melting temperatures (Tms) used for the SNaPshot assay.

### IFN-γ and IL-2 analysis

Baseline plasma levels of interferon-γ (IFN-γ) and interleukin-2 (IL-2) were measured to determine whether the patients were immunologically stable using an ELISA kit (R&D Systems, Minneapolis, MN, USA). To evaluate the changes in pharmacodynamic profiles caused by intracellular tacrolimus (i.e., immune reactivity), we performed flow cytometric assessment of IFN-γ and IL-2 in 39 patients, all of whom were randomly selected. PBMCs were cultured at a density of 1×10^6^ cells per 200 μL in 96-well round-bottom plates. The medium used was RPMI 1640 supplemented with 10% heat-inactivated fetal bovine serum and penicillin-streptomycin. PBMCs were activated with 50 ng/mL phorbol-12-myristate 13-acetate and 1 μg/mL ionomycin (Sigma-Aldrich, St. Louis, MO, USA) for 6 hours at 37°C with 5% CO_2_. To retain the cytokines within the cells, the protein transport inhibitor (BD GolgiStop^TM^; BD Biosciences, San Jose, CA, USA) was used beginning at 2 hours after activation. After stimulation, the cells were fixed and permeabilized with PBS and 0.5% Triton X-100. Subsequently, surface and intracellular staining of CD3-FITC, CD4-PE-Cyanine5, CD8-PE, and IFN-γ-APC (or IL-2-APC) (BD Biosciences, San Jose, CA, USA; eBioscience, San Diego, CA, USA) was performed. Isotype control and fluorescence minus one (FMO) control tubes were included as appropriate.

### Statistical analysis

All of the analyses and calculations were performed using SPSS (version 21.0; IBM, Armonk, NY, USA), STATA (version 12.0; StataCorp LP, College Station, TX, USA), and FlowJo (version 10.0.7; FlowJo LLC, Ashland, OR, USA). The data are presented as the mean ± standard deviation for continuous variables and as proportions for categorical variables. Based on histograms of the variable distributions, the variables with non-normal distributions are expressed as medians (interquartile ranges). The comparisons were evaluated using the chi-square test for categorical variables, ANOVA for parametric continuous variables (LSD post hoc analysis between two groups), and the Kruskal-Wallis test for non-parametric continuous variables (Mann-Whitney *U* test between two groups). The correlation coefficient between normally distributed continuous variables was measured using Pearson’s correlation test or the linear regression model. The fractional polynomials method was also applied to account for a possible nonlinear correlation. ANCOVA was used to determine the most significant parameter among multiple variables based on the hypothesis that all correlations were linear. The difference between paired samples with non-normal distributions was evaluated using the Wilcoxon signed-rank test. A *P* value of less than 0.05 was considered statistically significant.

## Results

### Baseline characteristics

A total of 214 patients were initially enrolled, but 1 patient had an IC-TAC less than the lower limit of quantification. Accordingly, 213 patients were finally analyzed. Table 1 shows the baseline characteristics of the study participants. All patients were of Asian descent. The WB-TAC, IC-TAC, and tacrolimus ratio were 4.6±1.83 ng/mL, 43.4±30.10 pg/10^6^ cells, and 9.3±4.25, respectively.

**Table 1:** Baseline characteristics of the study participants.

		Tacrolimus ratio (IC-TAC/WB-TAC)			
	Total (n = 213)	1^st^ quintile (n = 42)	2^nd^–4^th^ quintile (n = 129)	5^th^ quintile (n = 42)	*P*
Tacrolimus ratio	9.3 ± 4.25	5.1 ± 0.69^‡^	8.6 ± 1.61	15.7 ± 4.88^‡^	<0.001
Age (years)	47.1 ± 13.27	45.9 ± 13.18	47.9 ± 13.54	45.7 ± 13.27	0.538
Male sex (%)	59.2	78.6*	60.5	35.7^†^	<0.001
Donor source (%)					0.165
Living related donor	41.3	28.6	42.6	50.0	
Living unrelated donor	19.7	23.8	21.7	9.5	
Deceased donor	39.0	47.6	35.7	40.5	
Previous history of transplantation (%)	6.1	9.5	3.9	9.5	0.243
Diabetes mellitus (%)	16.9	19.0	19.4	7.1	0.170
Combined immunosuppressive agents					
Prednisolone (mg/day)	4.2 ± 1.57	4.3 ± 1.53	4.1 ± 1.63	4.5 ± 1.41	0.309
Mycophenolate mofetil (mg/day)	692.6 ± 464.84	701.9 ± 422.58	727.0 ± 472.02	577.6 ± 475.17	0.193
Blood findings					
Hematocrit (%)	40.9 ± 5.33	42.2 ± 6.72	41.1 ± 4.77	38.9 ± 4.97*	0.015
Lymphocyte (%)	29.8 ± 8.16	30.2 ± 7.42	30.0 ± 8.49	28.8 ± 7.96	0.672
Albumin (g/dL)	4.4 ± 0.30	4.5 ± 0.27*	4.3 ± 0.30	4.4 ± 0.32	0.088
Creatinine (mg/dL)	1.3 ± 0.45	1.27 ± 0.56	1.26 ± 0.39	1.25 ± 0.51	0.987
Proteinuria (%)	24.9	26.2	26.4	19.0	0.621
Delayed graft function (%)	3.8	7.1	3.9	0	0.226
Acute rejection (%)	15.6	9.5	18.0	14.3	0.410
Recurrence (%)	2.4	2.4	2.3	2.4	1.000
CIN (%)	7.5	4.8	8.5	7.1	0.720
Transplant duration (months)	58 (32–88)	63 (36–83)	59 (32–93)	50 (16–71)	0.140

Comparisons were evaluated using the chi-squared test for categorical variables, ANOVA for normally distributed continuous variables (LSD post hoc analysis between two groups), and the Kruskal-Wallis test for non-normally distributed continuous variables (Mann-Whitney *U* test between two groups). The 2^nd^ to 4^th^ quintile group served as a reference for comparison between two groups.

*P<0.05

^†^*P*<0.01

^‡^*P*<0.001.

IC-TAC, intracellular concentration of tacrolimus; WB-TAC, whole blood concentration of tacrolimus; CIN, calcineurin inhibitor-induced nephrotoxicity.

We analyzed three SNPs in the *ABCB1* gene (i.e., rs1045642, rs2032582, and rs1128503). Genotyping of *ABCB1* revealed that the major alleles of rs1045642, rs2032582, and rs1128503 were the C allele, G allele, and T allele, respectively, in the present cohort. The allele and genotype frequencies are described in S2 Table. All of these SNPs were within the Hardy-Weinberg equilibrium bounds (*P*s>0.05).

We also measured the baseline plasma levels of IFN-γ and IL-2 simultaneously, and found that most of the patients had undetectable levels of both cytokines (S1 Fig).

### Relationship between whole blood and intracellular tacrolimus concentrations

**Fig 1:**
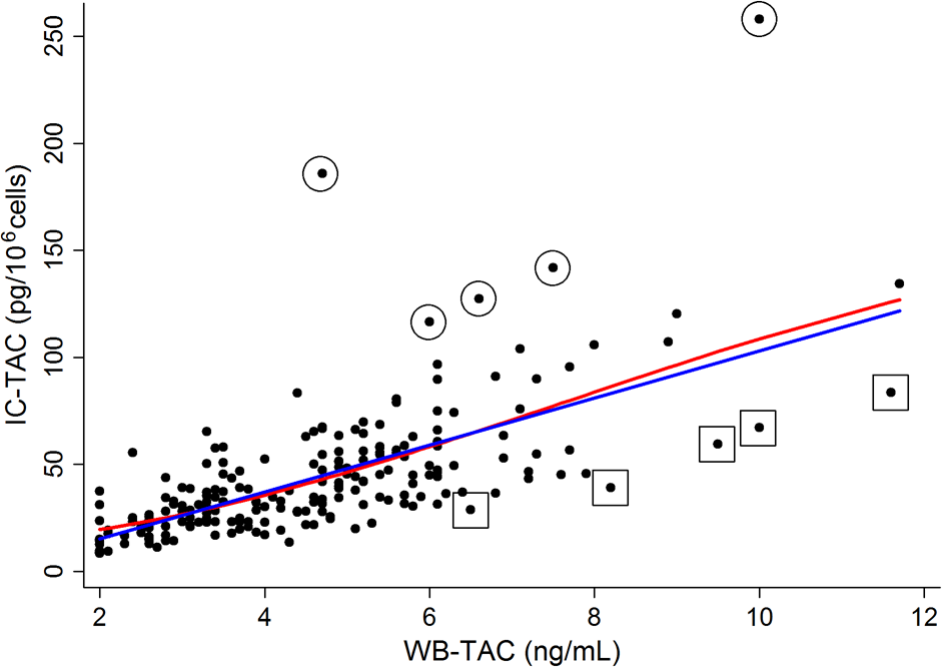
Scatter plot illustrating blood and intracellular tacrolimus concentrations. Blue and red lines represent linear and non-linear relationships between two variables, respectively. Circled dots indicate representative cases with high tacrolimus ratio; and squared dots indicate representative cases with low tacrolimus ratio. IC-TAC, intracellular concentration of tacrolimus; WB-TAC, whole blood concentration of tacrolimus.

**Fig 2:**
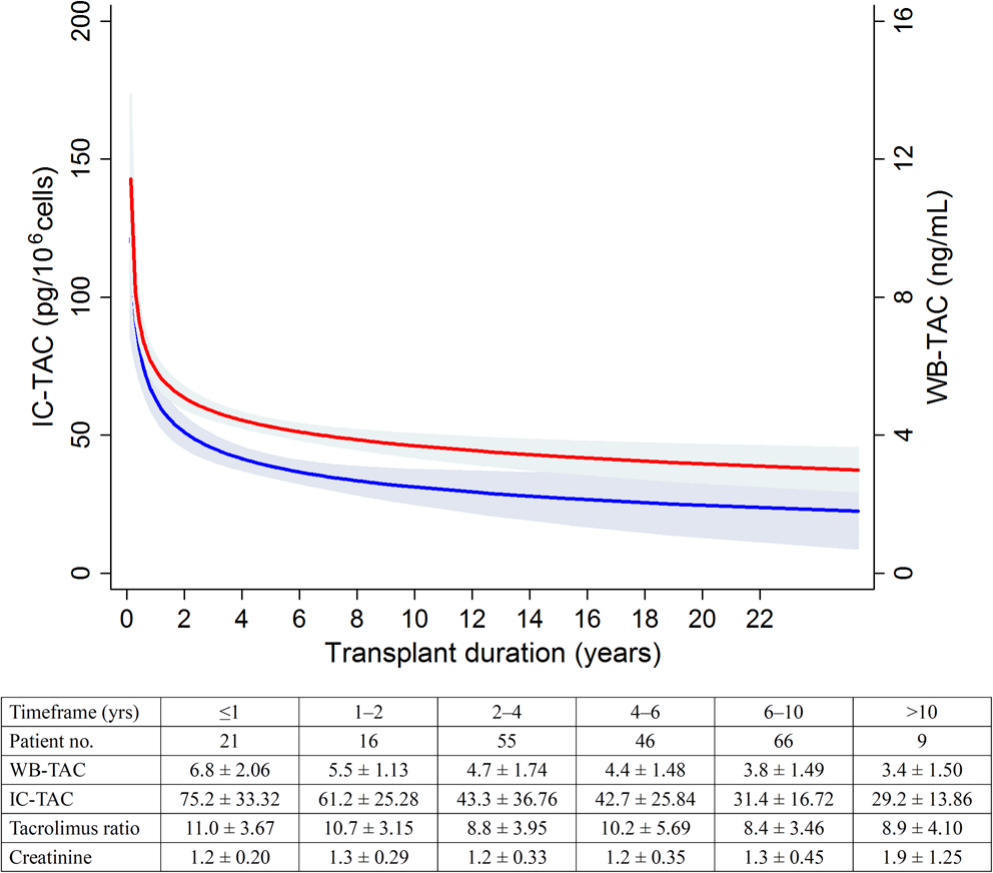
Dependence of the changes of IC-TAC (red line) and WB-TAC (blue line) on the transplant duration. The table below indicates the changes of tacrolimus parameters and graft function.

Fig 1 shows a scatter plot illustrating the distribution of the WB-TAC and IC-TAC. Pearson’s correlation coefficient (*r*) was 0.67 (*P*<0.001). When the non-linear method was applied (red line in Fig 1), the relationship also seemed to be linear. Nevertheless, we wanted to focus on the cases outside this correlation (i.e., outlier points of Fig 1). Accordingly, we defined the tacrolimus ratio (IC-TAC/WB-TAC) and sought to determine which cases were outside of this linear relationship (e.g., which characteristics do the circled or squared cases in Fig 1 have?). Patients were divided into three groups based on the quintiles of the tacrolimus ratio (Table 1). As a result, several factors such as sex, hematocrit, and albumin, were associated with the tacrolimus ratio. Table 2 shows the relationships after considering the tacrolimus ratio as a continuous variable: sex, hematocrit, and transplant duration were significantly correlated to the tacrolimus ratio. However, the SNPs in the *ABCB1* gene were not associated with the tacrolimus ratio except rs1045642 (S3 Table). Furthermore, most of the SNPs were not associated with the WB-TAC or IC-TAC, except for rs2032582 (considering the T allele as the major allele), which was associated with the WB-TAC. Histories of graft rejection, recurrence, or calcineurin inhibitor-induced nephrotoxicity were also not related with the tacrolimus ratio, even though we set up the timeframe to <2 weeks (data not shown). The multivariate model revealed that sex, hematocrit, and transplant duration were significant factors related to the tacrolimus ratio. However, other factors such as albumin and SNPs in the *ABCB1* gene were not independent factors. S4 Table provides detailed data obtained with the multivariate model (e.g., *F* and *P* values). Additionally, Fig 2 presents the trends of WB-TAC, IC-TAC, and tacrolimus ratio in each timeframe of transplantation.

**Table 2:** Baseline parameters associated with the ratio of intracellular tacrolimus level to blood tacrolimus level.

	Tacrolimus ratio (IC-TAC/WB-TAC)	Correlation coefficient	*P*
Age (years)^1^		-0.073	0.288
Sex^2^			
Male	8.3 ± 3.10		Reference
Female	10.7 ± 5.21		<0.001
Donor type^2^			
Living related donor	9.9 ± 4.61		Reference
Living unrelated donor	8.5 ± 2.81		0.083
Deceased donor	9.1 ± 4.41		0.216
History of transplantation^2^			
No	9.3 ± 4.29		Reference
Yes	9.0 ± 3.75		0.757
Diabetes mellitus^2^			
No	9.5 ± 4.49		Reference
Yes	8.4 ± 2.69		0.171
Immunosuppressive agents^1^			
Prednisolone (mg/day)		0.015	0.829
Mycophenolate mofetil (mg/day)		-0.070	0.311
Blood findings^1^			
Hematocrit (%)		-0.251	< 0.001
Lymphocyte (%)		-0.095	0.169
Albumin (g/dL)		-0.118	0.087
Creatinine (mg/dL)		-0.015	0.830
Proteinuria^2^			
No	9.2 ± 3.86		Reference
Yes	9.6 ± 5.30		0.587
Delayed graft function^2^			
No	9.4 ± 4.29		Reference
Yes	7.0 ± 2.30		0.122
Acute rejection^2^			
No	9.2 ± 4.28		Reference
Yes	9.7 ± 4.16		0.539
Recurrence^2^			
No	9.3 ± 4.24		Reference
Yes	10.0 ± 5.66		0.729
CIN^2^			
No	9.3 ± 4.34		Reference
Yes	9.2 ± 3.01		0.891
Transplant duration (years)^2^			
≤ 1	11.2 ± 3.57		Reference
1–2	10.4 ± 3.27		0.583
2–5	8.9 ± 3.75		0.031
> 5	9.1 ± 4.74		0.039

^1^Pearson’s correlation was used.

^2^Student’s t-test or post-hoc analysis (LSD) of ANOVA was used.

IC-TAC, intracellular concentration of tacrolimus; WB-TAC, whole blood concentration of tacrolimus; CIN, calcineurin inhibitor-induced nephrotoxicity.

Additionally, we explored the baseline characteristics according to the quintiles of IC-TAC (S4 Table). As a result, the transplant duration was the strongest factor affecting the IC-TAC levels. When multivariate analyses were performed (S5 Table), the transplant duration and the lymphocyte proportion remained significant in the relationship with IC-TAC.

**Fig 3:**
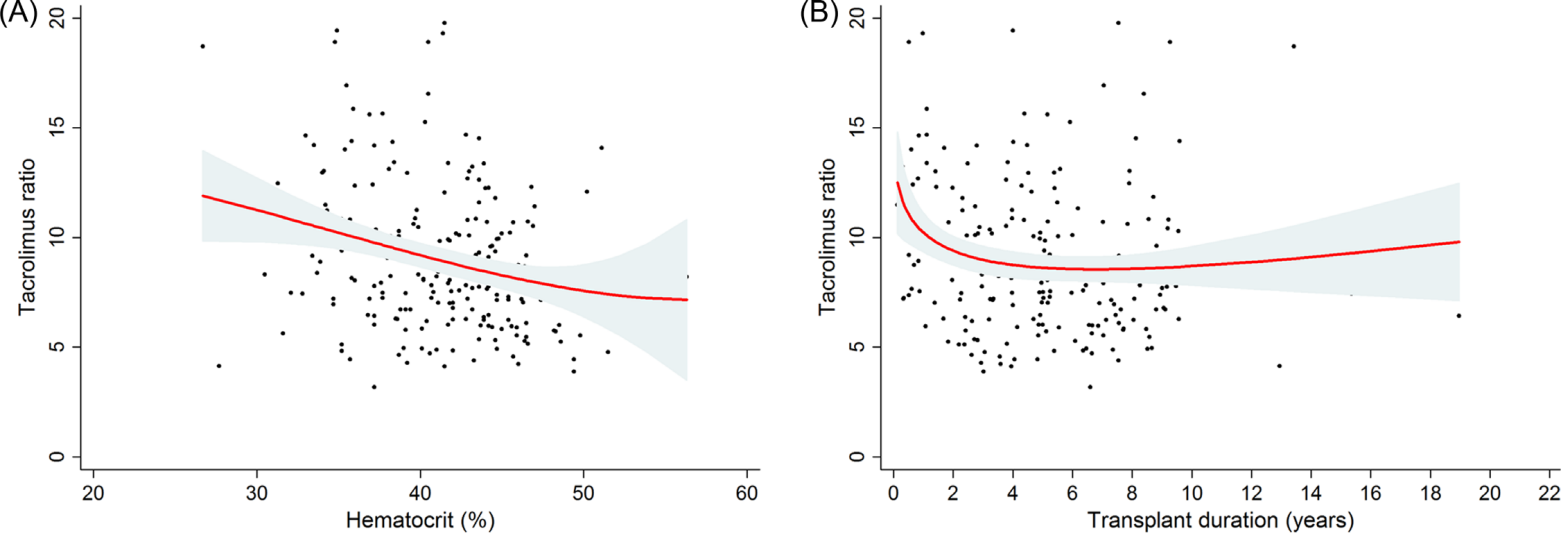
**Fitted curves between the tacrolimus ratio and the hematocrit (A) or transplant duration (B).** The range area indicates the 95% confidence interval.

**Fig 4:**
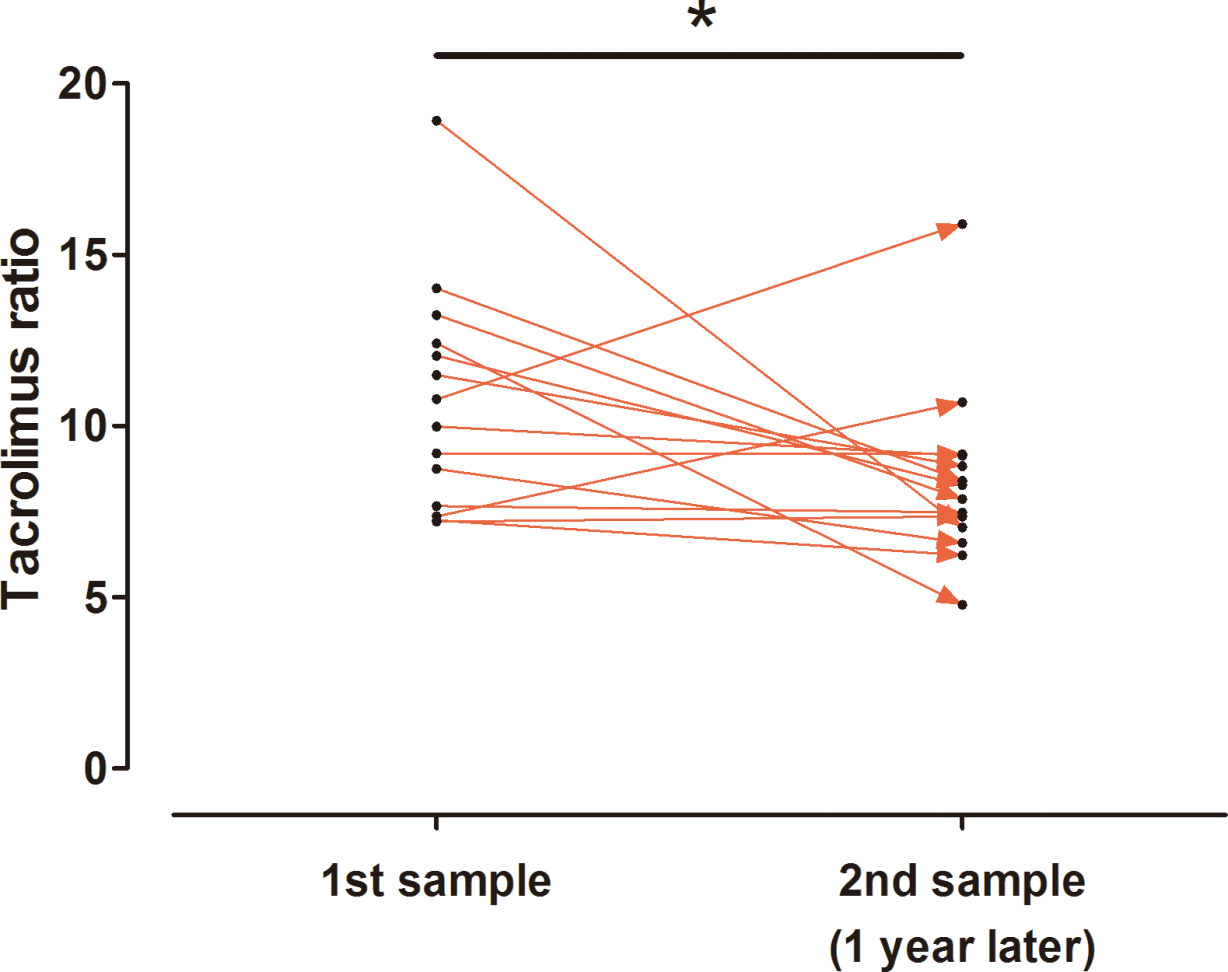
Change in the tacrolimus ratio between measurements taken one year apart. *P<0.05.

Subsequently, we assumed a potential non-linear relationship between these continuous parameters and the tacrolimus ratio (Fig 3). As a result, the relationship between hematocrit and the tacrolimus ratio appeared to be linear, but the relationship between transplant duration and the tacrolimus ratio appeared to be non-linear: the tacrolimus ratio decreased more abruptly in the early transplant period than in the later transplant period. With this in mind, we verified the change of tacrolimus ratio in 14 patients whose primary samples were obtained within 1 year of transplantation (Fig 4). After 1 year, secondary samples were obtained, and their tacrolimus ratios were significantly lower than those of the primary samples: 10.7 ± 3.30 in the primary samples vs. 8.4 ± 2.60 in the secondary samples; *P* = 0.041.

### Activation of T cells according to the intracellular tacrolimus concentration

**Fig 5:**
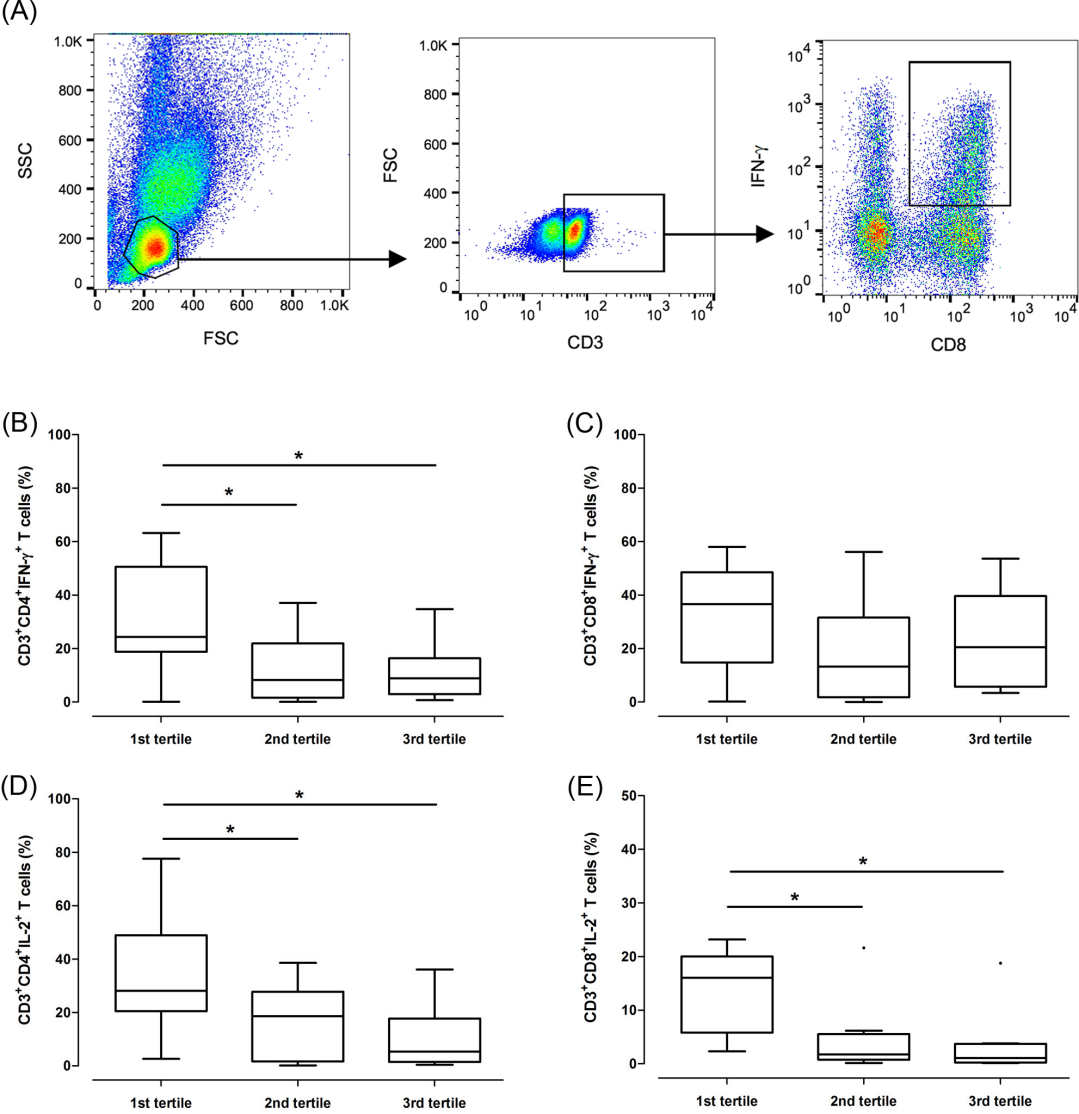
Activation of CD4^+^ (B and D) and CD8^+^ (C and E) T cells according to tertiles of intracellular tacrolimus concentrations. (A), Example of the dot plot gating strategy used to calculate the proportion of interferon-γ-producing CD3^+^CD8^+^ T cells. (B) and (C), Flow cytometry to identify T cells producing interferon-γ. (D) and (E), Flow cytometry to identify T cells producing interleukin-2. IFN-γ, interferon-γ; IL-2, interleukin-2.

We hypothesized that IC-TAC may have a clinical implication by predicting the immune reactivity as a potential risk of rejection [13]. With this in mind, we conducted flow cytometry of IFN-γ^+^ and IL-2^+^ T cells and compared the proportions of these cells with the IC-TAC tertiles (Fig 5). We found that the proportion of CD3^+^CD4^+^IFN-γ^+^ T cells was higher in the low-IC-TAC group than in the high-IC-TAC group. Similarly, CD3^+^CD4^+^IL-2^+^ and CD3^+^CD8^+^IL-2^+^ T cells were more abundant in the low-IC-TAC group than in the high-IC-TAC group. In contrast, the production of IFN-γ by CD8^+^ T cells was not associated with the IC-TAC. The overall trend in the WB-TAC was similar to that of the IC-TAC (S2 Fig). However, the correlations with CD3^+^CD8^+^IFN-γ^+^ and CD3^+^CD8^+^ IL-2^+^ T cells were not statistically significant for the WB-TAC.

## Discussion

A personalized approach to the use of tacrolimus is essential for improving graft outcome and reducing adverse events induced by tacrolimus. Recent studies have focused on this issue; however, the role of the intracellular tacrolimus concentration has not been thoroughly investigated. In the present study, we obtained three novel findings. First, we found that the correlation between blood and intracellular tacrolimus concentrations was relatively linear if the patients were stable. Second, sex, hematocrit, and transplant duration had significant effects on the balance between blood and intracellular tacrolimus concentrations, whereas SNPs in the *ABCB1* gene did not. Third, the IC-TAC determined the degree of T cell activation as a potential risk of rejection. In particular, the latter finding suggests that intracellular tacrolimus concentration monitoring may be reserved for a particular group of patients with an unbalance between IC-TAC and WB-TAC.

A few studies have been performed to examine the correlation between the WB-TAC and IC-TAC in transplant recipients [10,14,15]. The data on liver transplantation (n = 90) showed that the IC-TAC was not associated with the WB-TAC at 7 days [14]. Unlike the WB-TAC, the IC-TAC had a significant association with quantitative scores related to hepatic histology and clinical rejection. Based on the heart transplant data (28 samples from 24 patients) [15], the IC-TAC was not related to the WB-TAC, and the median duration of transplantation was 1255 days (range: 65 days to 9556 days). The previous kidney transplant data (from 96 patients) also revealed that there was no correlation between the IC-TAC and WB-TAC at 7 days or at the steady state (detailed descriptions of the sample number in each timeframe were not provided, and no data on the timing of the steady state were presented) [10]. In contrast to those studies, our data showed that the IC-TAC had a relatively linear correlation with the WB-TAC, although the relationship was not perfect. These conflicting results may be due to the fact that all of the previous studies had small or modest sample sizes and did not evaluate whether the patients were systemically stable. The examination of patients for only 7 days after transplantation is insufficient to guarantee that both the graft and patient are stable. We included only patients with stable graft functions and further confirmed that they had low levels of inflammatory cytokines. Nevertheless, we cannot suggest that the IC-TAC completely corresponded with the WB-TAC. As shown in the markers of Fig 1, our primary focus was outlying cases outside the linear correlation. Accordingly, we attempted to identify the factors related to the balance between the two compartments (i.e., blood and lymphocyte) by defining the tacrolimus ratio.

Sex-dependent differences in the pharmacokinetics of tacrolimus have been reported [16]. These differences may be attributable to complex interactions among genetic, hormonal, and socio-economic factors [17]. However, the currently available information is limited to the blood level, as studies have not been performed to examine the cellular level. We initially found that female patients had higher IC-TACs than male patients, even when their blood tacrolimus concentrations were the same. Although we could not determine the mechanism underlying this result, this finding provides support for previous studies wherein female patients exhibited lower WB-TACs than male patients after being administered the same dose of tacrolimus; in females, intracellular tacrolimus can be effluxed from the cells, causing greater increases in blood levels compared with the levels observed in males.

Additionally, we found that both the hematocrit and the transplant duration were negatively associated with the tacrolimus ratio. The hematocrit determines the amount of tacrolimus that exists in an unbound form because of saturable binding to red blood cells [18]. With this in mind, a large unbound fraction of tacrolimus due to a low hematocrit can induce an influx of tacrolimus into the intracellular compartment and increase the tacrolimus ratio. Regarding transplant duration, the tacrolimus ratio in the early transplant period was significantly higher than that in the later period. Furthermore, the correlation appeared to be non-linear: changes in the tacrolimus ratio were observed primarily in the early transplant period. Our paired sample test for the subsequent samples (i.e., one year apart) verified this result. This finding indicates that the decrease in the IC-TAC would be largely non-proportional to the decrease in the WB-TAC during the early period. A potential underlying mechanism could not be identified; however, this finding has clinical implications, particularly when tacrolimus must be tapered. We revealed that the IC-TAC had a larger impact on the activation of T cells than the WB-TAC, and this information suggests that monitoring the WB-TAC and IC-TAC together, especially in the early period, can be valuable in preventing rejection during the tapering period compared with monitoring the WB-TAC alone.

Tacrolimus is a substrate of ABCB1, which has an excretory function [19]. This protein is also expressed in lymphocytes and may play a role in viral resistance and in trafficking cytokines and enveloped viruses [20]. It is plausible that the expression or function of ABCB1 affects the IC-TAC. However, in contrast to previous study results [10], we could not identify an independent association between any SNP in *ABCB1* and the tacrolimus ratio (or the IC-TAC). This result may be due to several factors. Primarily, the associations between SNPs of *ABCB1* and molecular or clinical phenotypes have been largely inconsistent across several studies [21], and SNPs do not necessarily affect the expression or function of the *ABCB1* gene. Furthermore, various external stresses, such as recipient and donor factors, could alter the expression or function of ABCB1. Additionally, previous studies focused mainly on short-term periods and did not adjust for other covariates such as sex, hematocrit, and transplant duration [10]. Compared with the previous studies, the present study considered many factors on this issue.

Although the present results are informative, this study has a limitation. The cross-sectional study design did not provide information on the clinical outcomes (e.g., rejection) according to the IC-TAC, and thus the clinical implication of IC-TAC may not be supported by our results alone. However, the present study measured patients’ T cell activation as a marker of their immune status, because the monitoring of immune reactivity is known to be related with the risk of rejection [13]. Accordingly, IC-TAC was well correlated with the degree of T cell reactivity. For the WB-TAC, the correlation was also consistent, but modest compared with IC-TAC. Nevertheless, the current study design restricted further exploration of the significance of IC-TAC because each patient had a different transplantation timeframe. IC-TAC would be most relevant with transplant outcome, as intracellular tacrolimus causes the biologic effect within targets (i.e., lymphocytes). To solve these problems, studies including patients with the same transplant period are recommended.

Appropriate use of tacrolimus in kidney transplant recipients is essential because it can reduce the current risks of rejection and adverse events triggered by tacrolimus. We may need information about the intracellular tacrolimus concentrations in particular cases affecting the balance of tacrolimus between the blood and intracellular compartments. The present results addressed this issue only in the patients with stable graft functions. Accordingly, future studies will determine the issues, such as values in the rejection state or other unstable conditions before a clinical application of intracellular tacrolimus monitoring could become available.

## Supporting Information

S1 Fig **Plasma levels of interferon-γ (A) and interleukin-2 (B) in the study participants at the time of tacrolimus concentration measurement.** Most of the patients had low levels of these cytokines. IFN-γ, interferon-γ; IL-2, interleukin-2.
(TIF)

S2 Fig **Activation of CD4**^**+**^ **(A and C) or CD8**^**+**^ **(B and D) T cells according to tertiles of whole blood tacrolimus concentrations.** (A) and (B), Flow cytometry to identify T cells producing interferon-γ. (C) and (D), Flow cytometry to identify T cells producing interleukin-2. IFN-γ, interferon-γ; IL-2, interleukin-2.
(JPG)

S1 Instrumentation for LC-MS/MS (DOC)

S1 Table SNP frequencies in kidney transplant recipients. (DOC)

S2 Table Association between SNP of *ABCB1* and tacrolimus concentration. (DOC)

S3 Table Analysis of covariance with the assumption that the relationship with the tacrolimus ratio is linear. (DOC)

S4 Table Baseline characteristics according to the intracellular tacrolimus concentrations. (DOC)

S5 Table Analysis of covariance with the assumption that the relationship with the intracellular tacrolimus concentration is linear. (DOC)
