# Supplementary figures and images for: Monitoring the Intracellular Tacrolimus Concentration in Kidney Transplant Recipients with Stable Graft Function
Source: PLoS One. 2016 Apr 15;11(4):e0153491. doi: 10.1371/journal.pone.0153491 (PMC4833335; doi:10.1371/journal.pone.0153491)

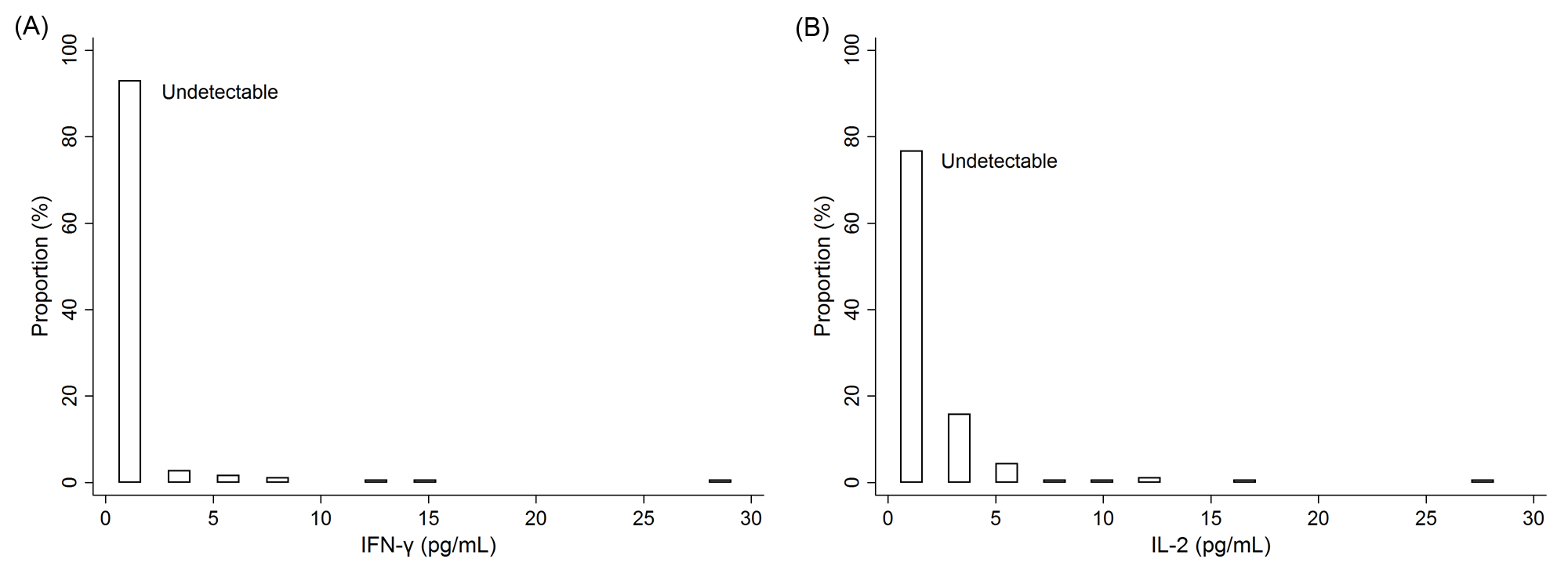

Supplement: S1 Fig — Plasma levels of interferon-γ (A) and interleukin-2 (B) in the study participants at the time of tacrolimus concentration measurement. Most of the patients had low levels of these cytokines. IFN-γ, interferon-γ; IL-2, interleukin-2. (TIF) [file pone.0153491.s001.tif]

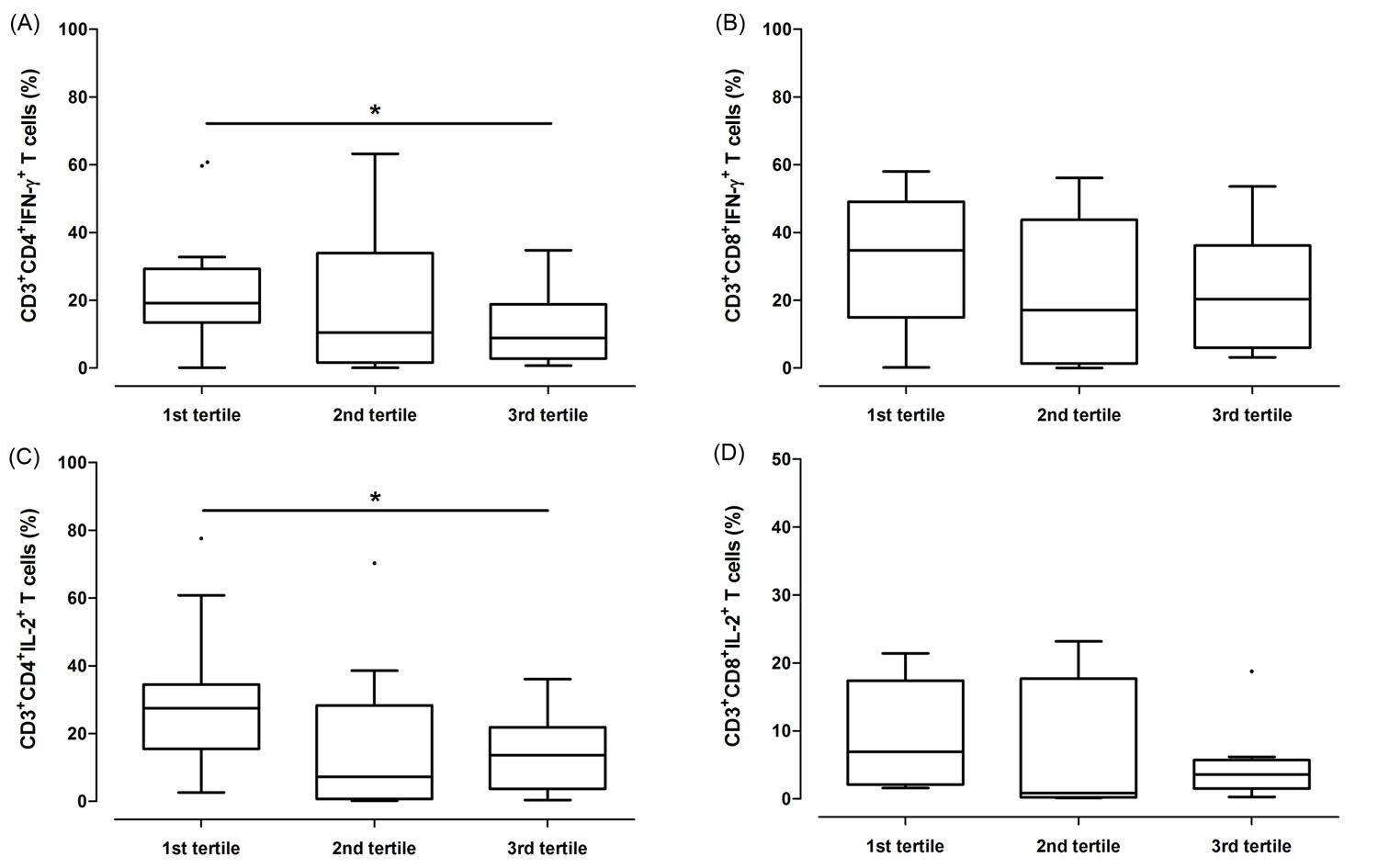

Supplement: S2 Fig — Activation of CD4+ (A and C) or CD8+ (B and D) T cells according to tertiles of whole blood tacrolimus concentrations. (A) and (B), Flow cytometry to identify T cells producing interferon-γ. (C) and (D), Flow cytometry to identify T cells producing interleukin-2. IFN-γ, interferon-γ; IL-2, interleukin-2. (JPG) [file pone.0153491.s002.jpg]
