# Supplementary material for: Monitoring the Intracellular Tacrolimus Concentration in Kidney Transplant Recipients with Stable Graft Function
Source: PLoS One. 2016 Apr 15;11(4):e0153491. doi: 10.1371/journal.pone.0153491 (PMC4833335; doi:10.1371/journal.pone.0153491)
Supplement: S1 Instrumentation for LC-MS/MS — (DOC) [file pone.0153491.s003.doc]

S1 Instrumentation for LC-MS/MS

#1. HPLC: Agilent 1260 Infinity Binary LC system (Agilent Technologies, Santa Clara, CA, USA)

| Analytical column | Venusil XBP C_18_ (50 × 2.1 mm, 5 µm, Agela Technologies, Newark, NJ, USA) |
| --- | --- |
| Column temperature | 40°C |
| Mobile phase | A) 0.1% formic acid in 2 mM ammonium acetate; B) 0.1% formic acid in 100% methanol |
| Flow rate | 0.2 mL/min |
| Injection volume | 5 µL |
| Autosampler temperature | 4°C |
| Run time | 5 minutes |
| Retention time | Tacrolimus, 1.02 minutes; IS, 1.01 minutes |

#2. MS/MS: API 4000 Qtrap (AB Sciex, Framingham, MA, USA)

| Ion source | ESI, positive |
| --- | --- |
| Scan type | MRM mode |
| Parameter | CAD Medium, CUR 20, GS1 50, GS2 50, IS 5500, TEM 500 |
