# Supplementary material for: Monitoring the Intracellular Tacrolimus Concentration in Kidney Transplant Recipients with Stable Graft Function
Source: PLoS One. 2016 Apr 15;11(4):e0153491. doi: 10.1371/journal.pone.0153491 (PMC4833335; doi:10.1371/journal.pone.0153491)
Supplement: S1 Table — (DOC) [file pone.0153491.s004.doc]

S1 Table. SNP frequencies in kidney transplant recipients

|  | Allele frequency | | Genotype frequency | |  |
| --- | --- | --- | --- | --- | --- |
| SNP position | Allele | N (%) | Genotype | N (%) | *P* |
| rs1045642 | C | 280 (65.7) | C/C | 89 (41.8) | 0.656 |
|  | T | 146 (34.3) | C/T | 102 (47.9) |  |
|  |  |  | T/T | 22 (10.3) |  |
| rs2032582 | G | 214 (50.2) | G/G | 46 (21.6) | 0.105 |
|  | O | 212 (49.8) | G/O | 122 (57.3) |  |
|  |  |  | O/O | 45 (21.1) |  |
|  | A | 70 (16.4) | A/A | 4 (1.9) | 0.683 |
|  | P | 356 (83.6) | A/P | 62 (29.1) |  |
|  |  |  | P/P | 147 (69.0) |  |
|  | T | 142 (33.3) | T/T | 20 (9.4) | 0.528 |
|  | Q | 284 (66.7) | T/Q | 102 (47.9) |  |
|  |  |  | Q/Q | 91 (42.7) |  |
| rs1128503 | C | 186 (43.7) | C/C | 39 (18.3) | 0.905 |
|  | T | 240 (56.3) | C/T | 108 (50.7) |  |
|  |  |  | T/T | 66 (31.0) |  |

O, P, and Q are counterpart alleles for G, A, and T, respectively.
