# Supplementary material for: Monitoring the Intracellular Tacrolimus Concentration in Kidney Transplant Recipients with Stable Graft Function
Source: PLoS One. 2016 Apr 15;11(4):e0153491. doi: 10.1371/journal.pone.0153491 (PMC4833335; doi:10.1371/journal.pone.0153491)
Supplement: S2 Table — (DOC) [file pone.0153491.s005.doc]

S2 Table. Association between SNP of *ABCB1* and tacrolimus concentration

|  |  | WB-TAC | | IC-TAC | | IC-TAC/WB-TAC | |
| --- | --- | --- | --- | --- | --- | --- | --- |
| SNP position | Genotype | Concentration (ng/mL) | *P* | Concentration (pg/10^6^ cells) | *P* | Ratio (pg.10^-6^ cells/ng.mL^-1^) | *P* |
| rs1045642 | C/C^1^ | 4.5 ± 1.82 | 0.464 | 42.1 ± 27.04 | 0.345 | 8.9 ± 3.39 | 0.111 |
|  | C/T | 4.5 ± 1.70 |  | 42.5 ± 30.89 |  | 9.3 ± 3.99 |  |
|  | T/T | 5.0 ± 2.39 |  | 52.2 ± 37.45 |  | 11.1 ± 7.35* |  |
| rs2032582 | G/G^1^ | 4.6 ± 2.07 | 0.204 | 45.7 ± 31.28 | 0.164 | 9.4 ± 3.34 | 0.315 |
|  | G/O | 4.4 ± 1.60 |  | 40.2 ± 28.74 |  | 9.0 ± 4.01 |  |
|  | O/O | 5.0 ± 2.09 |  | 49.7 ± 31.93 |  | 10.1 ± 5.52 |  |
|  | A/A^1^ | 4.3 ± 1.01 | 0.820 | 41.8 ± 11.91 | 0.903 | 10.0 ± 2.69 | 0.619 |
|  | A/P | 4.7 ± 1.67 |  | 42.0 ± 23.36 |  | 8.9 ± 3.36 |  |
|  | P/P | 4.6 ± 1.83 |  | 43.4 ± 30.10 |  | 9.5 ± 4.61 |  |
|  | T/T^1^ | 5.5 ± 2.35 | 0.032 | 54.5 ± 38.61 | 0.215 | 10.6 ± 7.63 | 0.318 |
|  | T/Q | 4.3 ± 1.69* |  | 41.7 ± 31.20 |  | 9.4 ± 4.08 |  |
|  | Q/Q | 4.6 ± 1.80 |  | 43.4 ± 30.10 |  | 9.0 ± 3.34 |  |
| rs1128503 | C/C^1^ | 4.4 ± 1.48 | 0.429 | 40.1 ± 21.39 | 0.164 | 9.1 ± 3.69 | 0.211 |
|  | C/T | 4.5 ± 1.71 |  | 41.0 ± 25.23 |  | 8.9 ± 3.35 |  |
|  | T/T | 4.8 ± 2.17 |  | 49.2 ± 39.88 |  | 10.1 ± 5.62 |  |

^1^This group served as a reference for comparison between two groups.

**P* <0.05.

O, P, and Q are counterpart alleles for G, A, and T, respectively.
