# Supplementary material for: Monitoring the Intracellular Tacrolimus Concentration in Kidney Transplant Recipients with Stable Graft Function
Source: PLoS One. 2016 Apr 15;11(4):e0153491. doi: 10.1371/journal.pone.0153491 (PMC4833335; doi:10.1371/journal.pone.0153491)
Supplement: S3 Table — (DOC) [file pone.0153491.s006.doc]

S3 Table. Analysis of covariance with the assumption that the relationship with the tacrolimus ratio is linear

| Parameter | df | Mean square | *F* | *P* |
| --- | --- | --- | --- | --- |
| Age | 1 | 20.932 | 1.275 | 0.260 |
| Sex | 1 | 83.933 | 5.111 | 0.025 |
| Donor type | 2 | 16.550 | 1.008 | 0.367 |
| History of transplantation | 1 | 10.824 | 0.659 | 0.418 |
| Diabetes mellitus | 1 | 28.693 | 1.747 | 0.188 |
| Prednisolone | 1 | 26.474 | 1.612 | 0.206 |
| Mycophenolate mofetil | 1 | 0.636 | 0.039 | 0.844 |
| Hematocrit | 1 | 75.193 | 4.579 | 0.034 |
| Lymphocyte | 1 | 54.688 | 3.330 | 0.070 |
| Albumin | 1 | 20.836 | 1.269 | 0.261 |
| Creatinine | 1 | 9.184 | 0.559 | 0.456 |
| Proteinuria | 1 | 1.714 | 0.104 | 0.747 |
| Delayed graft function | 1 | 34.265 | 2.087 | 0.150 |
| Acute rejection | 1 | 19.438 | 1.184 | 0.278 |
| Recurrence | 1 | 8.402 | 0.512 | 0.475 |
| Calcineurin inhibitor-induced nephrotoxicity | 1 | 0.368 | 0.022 | 0.881 |
| Transplant duration | 1 | 118.780 | 7.233 | 0.008 |
| rs1045642 | 2 | 3.211 | 0.196 | 0.823 |
| rs2032582 | 2 | 3.456 | 0.210 | 0.810 |
| rs1128503 | 2 | 0.330 | 0.020 | 0.980 |
