# Supplementary material for: Monitoring the Intracellular Tacrolimus Concentration in Kidney Transplant Recipients with Stable Graft Function
Source: PLoS One. 2016 Apr 15;11(4):e0153491. doi: 10.1371/journal.pone.0153491 (PMC4833335; doi:10.1371/journal.pone.0153491)
Supplement: S4 Table — (DOC) [file pone.0153491.s007.doc]

S4 Table. Baseline characteristics according to the intracellular tacrolimus concentrations

|  | Intracellular tacrolimus concentrations | | | |
| --- | --- | --- | --- | --- |
|  | 1^st^ quintile (n=42) | 2^nd^–4^th^ quintile (n=129) | 5^th^ quintile (n=42) | *P* |
| Intracellular tacrolimus concentration (pg/10^6^ cells) | 16.8 ± 3.98 | 37.5 ± 10.31^‡^ | 87.8 ± 38.52^‡^ | <0.001 |
| Age (years) | 47.4 ± 14.70 | 47.3 ± 13.42 | 46.0 ± 11.47 | 0.856 |
| Male sex (%) | 69.0 | 59.7 | 47.6 | 0.133 |
| Donor source (%) |  |  |  | 0.080 |
| Living related donor | 31.0 | 42.6 | 47.6 |  |
| Living unrelated donor | 16.7 | 24.0 | 9.5 |  |
| Deceased donor | 52.4 | 33.3 | 42.9 |  |
| Previous history of transplantation (%) | 4.8 | 5.4 | 9.5 | 0.579 |
| Diabetes mellitus (%) | 19.0 | 16.3 | 16.7 | 0.916 |
| Combined immunosuppressive agents |  |  |  |  |
| Prednisolone (mg/day) | 4.4 ± 1.56 | 3.9 ± 1.53 | 4.8 ± 1.51^†^ | 0.002 |
| Mycophenolate mofetil (mg/day) | 667.6 ± 482.70 | 737.4 ± 447.91 | 579.8 ± 457.88 | 0.150 |
| Blood findings |  |  |  |  |
| Hematocrit (%) | 40.4 ± 5.55 | 41.3 ± 5.57 | 40.0 ± 4.19 | 0.369 |
| Lymphocyte (%) | 29.3 ± 8.06 | 30.6 ± 7.95 | 27.7 ± 8.67* | 0.114 |
| Albumin (g/dL) | 4.4 ± 0.31 | 4.4 ± 0.27 | 4.4 ± 0.38 | 0.883 |
| Creatinine (mg/dL) | 1.30 ± 0.57 | 1.26 ± 0.40 | 1.23 ± 0.46 | 0.771 |
| Proteinuria (%) | 35.7 | 24.0 | 16.7 | 0.122 |
| Delayed graft function (%) | 4.8 | 4.7 | 0 | 0.360 |
| Acute rejection (%) | 9.5 | 20.2 | 7.3 | 0.069 |
| Recurrence (%) | 2.4 | 1.6 | 4.9 | 0.473 |
| CIN (%) | 4.8 | 7.8 | 9.5 | 0.700 |
| Transplant duration (months) | 79 (48–97) | 59 (34–87)* | 27 (10–53)^‡^ | <0.001 |

Comparisons were evaluated using the chi-squared test for categorical variables, ANOVA for normally distributed continuous variables (LSD post hoc analysis between two groups), and the Kruskal-Wallis test for non-normally distributed continuous variables (Mann-Whitney *U* test between two groups). The 2^nd^ to 4^th^ quintile group served as a reference for comparison between two groups.

**P*<0.05; ^†^*P*<0.01; ^‡^*P*<0.001.

IC-TAC, intracellular concentration of tacrolimus; WB-TAC, whole blood concentration of tacrolimus; CIN, calcineurin inhibitor-induced nephrotoxicity.
