# Supplementary material for: Monitoring the Intracellular Tacrolimus Concentration in Kidney Transplant Recipients with Stable Graft Function
Source: PLoS One. 2016 Apr 15;11(4):e0153491. doi: 10.1371/journal.pone.0153491 (PMC4833335; doi:10.1371/journal.pone.0153491)
Supplement: S5 Table — (DOC) [file pone.0153491.s008.doc]

S5 Table. Analysis of covariance with the assumption that the relationship with the intracellular tacrolimus concentration is linear

| Parameter | df | Mean square | *F* | *P* |
| --- | --- | --- | --- | --- |
| Age | 1 | 1053..451 | 1.380 | 0.242 |
| Sex | 1 | 1639.650 | 2.147 | 0.145 |
| Donor type | 2 | 140.026 | 0.183 | 0.833 |
| History of transplantation | 1 | 909.172 | 1.191 | 0.277 |
| Diabetes mellitus | 1 | 867.214 | 1.136 | 0.288 |
| Prednisolone | 1 | 149.093 | 0.195 | 0.659 |
| Mycophenolate mofetil | 1 | 1051.419 | 1.377 | 0.242 |
| Hematocrit | 1 | 935.636 | 1.225 | 0.270 |
| Lymphocyte | 1 | 3051.414 | 3.996 | 0.047 |
| Albumin | 1 | 864.261 | 1.132 | 0.289 |
| Creatinine | 1 | 1629.428 | 2.134 | 0.146 |
| Proteinuria | 1 | 374.748 | 0.491 | 0.484 |
| Delayed graft function | 1 | 1238.454 | 1.622 | 0.204 |
| Acute rejection | 1 | 20.876 | 0.027 | 0.869 |
| Recurrence | 1 | 1.280 | 0.002 | 0.967 |
| Calcineurin inhibitor-induced nephrotoxicity | 1 | 3.918 | 0.005 | 0.943 |
| Transplant duration | 1 | 16277.977 | 21.318 | <0.001 |
| rs1045642 | 2 | 44.769 | 0.059 | 0.943 |
| rs2032582 | 2 | 63.005 | 0.083 | 0.921 |
| rs1128503 | 2 | 352.437 | 0.462 | 0.631 |
